# A Treatise on Practical Anatomy

**Published:** 1891-12

**Authors:** 


A Treatise on Practical Anatomy; For Students of Anatomy and Surgery. By Henry C. Bœnning, M. D., Lecturer on Anatomy and Surgery in the Philadelphia School of Anatomy, etc., etc. Illustrated with 198 wood-engravings. Philadelphia and London: F. A. Davis, Publisher, 1891. Price, $2.50 net.

The student of Anatomy who buys this excellent book, makes no mistake. Devoid of all superfluous verbiage, it is to the point. Fully illustrated throughout with about 200 engravings, the work compares well with other works of like character. It is a pleasure to turn over the leaves and find at a glance just what you need, just what you are looking for. It is a beautiful and handsome octavo volume, printed in extra large, clear type, making it specially desirable for use in the dissecting room. It is substantially bound in extra cloth, with nearly 500 pages, and is a credit to author and publisher alike.

Professor Bœnning may be assured of the thanks of the whole profession for this superb treatise. W. S.

